# Recent advances in photothermal nanomaterials for ophthalmic applications

**DOI:** 10.3762/bjnano.16.16

**Published:** 2025-02-17

**Authors:** Jiayuan Zhuang, Linhui Jia, Chenghao Li, Rui Yang, Jiapeng Wang, Wen-an Wang, Heng Zhou, Xiangxia Luo

**Affiliations:** 1 Clinical College of Chinese Medicine, Gansu University of Chinese Medicine, Lanzhou 730000, P. R. China; 2 School of Public Health, Yangzhou University, Yangzhou 225009, P. R. China; 3 School of Marine Science and Engineering, Hainan University, Haikou 570228, P. R. China; 4 Medical College, Yangzhou University, Yangzhou 225009, P. R. China; 5 The first school of clinical medicine, Lanzhou University, Lanzhou 730000, P. R. China; 6 Gansu Provincial Hospital of TCM, Lanzhou 730000, P. R. China

**Keywords:** multifunctional composite, ophthalmology, photothermal nanomaterial, thermal field distribution, vapor nanobubble

## Abstract

The human eye, with its remarkable resolution of up to 576 million pixels, grants us the ability to perceive the world with astonishing accuracy. Despite this, over 2 billion people globally suffer from visual impairments or blindness, primarily because of the limitations of current ophthalmic treatment technologies. This underscores an urgent need for more advanced therapeutic approaches to effectively halt or even reverse the progression of eye diseases. The rapid advancement of nanotechnology offers promising pathways for the development of novel ophthalmic therapies. Notably, photothermal nanomaterials, particularly well-suited for the transparent tissues of the eye, have emerged as a potential game changer. These materials enable precise and controllable photothermal therapy by effectively manipulating the distribution of the thermal field. Moreover, they extend beyond the conventional boundaries of thermal therapy, achieving unparalleled therapeutic effects through their diverse composite structures and demonstrating enormous potential in promoting retinal drug delivery and photoacoustic imaging. This paper provides a comprehensive summary of the structure–activity relationship between the photothermal properties of these nanomaterials and their innovative therapeutic mechanisms. We review the latest research on photothermal nanomaterial-based treatments for various eye diseases. Additionally, we discuss the current challenges and future perspectives in this field, with a focus on enhancing global visual health.

## Review

### 1 Introduction

The human eye, serving as a primary organ for information acquisition, is vulnerable to various diseases over a lifetime [1]. The Global Sight Database reported that, in 2020, the world had 43.3 million individuals suffering from blindness and an additional 295 million with moderate to severe visual impairments [2]. The past decade has seen notable advancements in ophthalmic treatment techniques, including the widespread clinical adoption of anti-vascular endothelial growth factor drugs and laser therapy [3–4]. Despite these strides, challenges remain in effectively addressing complex ophthalmic issues, particularly in underprivileged areas where high costs associated with precision optical diagnostics and advanced drug treatments pose significant barriers [5].

The evolution of nanotechnology has catalyzed the development of novel therapeutic technologies, with a plethora of nanomaterials exhibiting significant potential for nanotherapeutic applications [6–8]. Among these, photothermal nanomaterials hold promise in ophthalmology because of their compatibility with the high transmittance characteristics of ocular tissues, allowing for a more sensitive response to various types of incident light [9–11]. This sensitivity facilitates diverse therapeutic effects and offers solutions to complex ophthalmic diseases. In addition, the cornea, an ocular tissue, is relatively “immune amnestied” because of the absence of blood vessels and lymphatic vessels, which reduces the patient’s immune response and inflammation and improves the safety and efficacy of photothermal nanomaterials therapy [12–13]. The small size of photothermal nanomaterials enhances their ability to penetrate the blood–ocular barrier, ensuring more precise control over thermal field distribution, thereby reducing the need for high optical power and improving safety [14]. The functional versatility of photothermal nanomaterials, attributable to their rich functional groups and surface dangling bonds, enables the effective loading of drugs, targeting molecules, and antibodies [15]. When combined with thermal/pH-sensitive materials, shape memory materials, and hydrogels, they form an efficient platform for photothermal therapy [16]. The efficient photothermal conversion and tunability of light absorption of these materials simplify the therapeutic light source, broadening their applicability [17–18]. Furthermore, the customizable nature of these nanomaterials allows for the development of personalized treatment plans, tailored to individual patient conditions [19–21].

**Figure 1:**
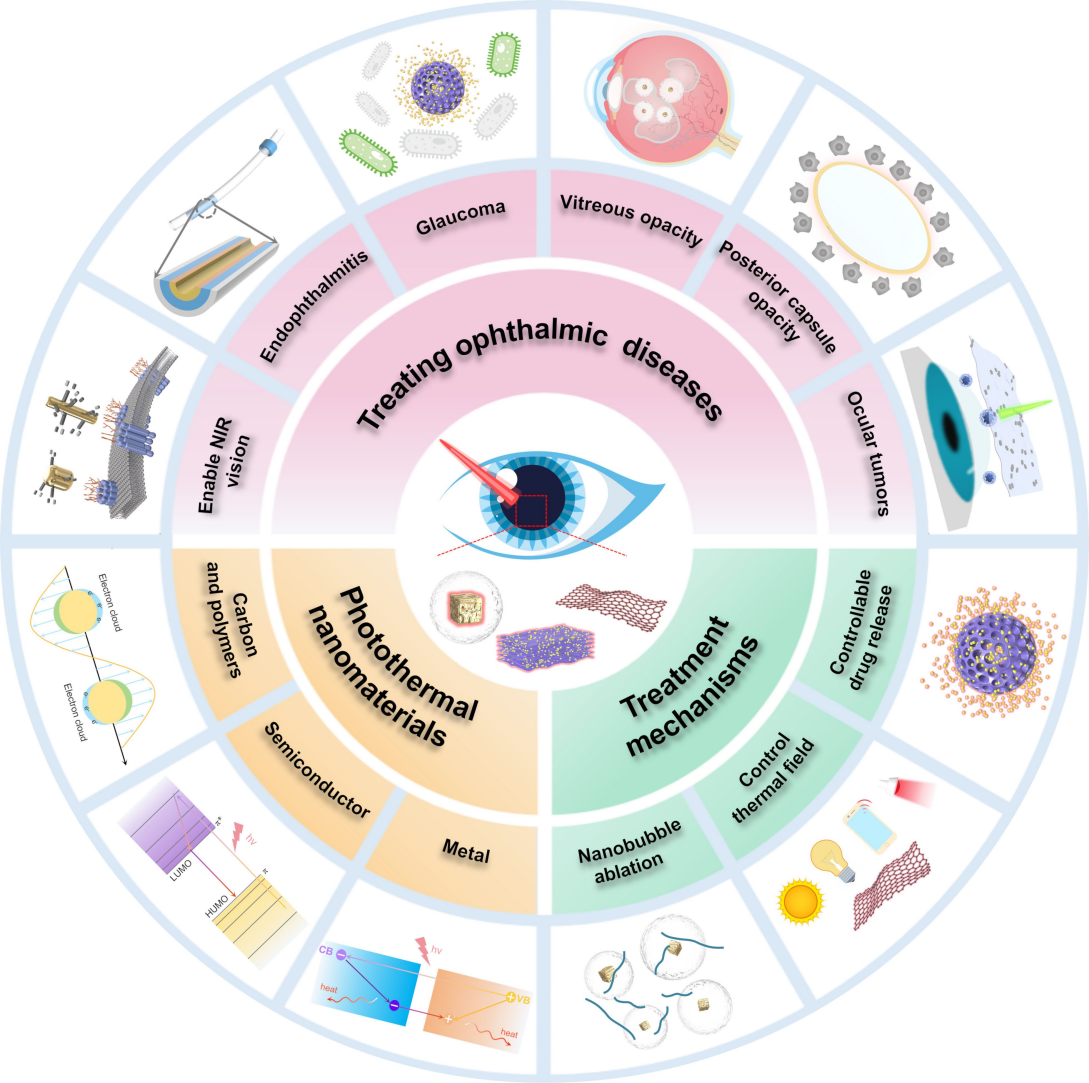
Overview of ophthalmic treatment technologies based on photothermal nanomaterials.

The straightforward and cost-effective fabrication of photothermal nanomaterials enhances their practicality, promising rapid advancements in the field of photothermal nanotherapeutics in ophthalmology. This paper reviews recent research progress in the application of photothermal nanomaterials for treating various ophthalmic diseases, including ocular tumors, glaucoma, cataracts, vitreous opacity, endophthalmitis, and decreased visual acuity. It also summarizes the structure–activity relationships between the photothermal properties of these materials and novel therapeutic mechanisms (Figure 1). In addition, the application progress of photothermal nanomaterials in promoting retinal drug delivery and enhancing photoacoustic imaging was also discussed. Finally, we address the current challenges and prospects in ophthalmic treatment technologies based on photothermal nanomaterials, with an aim to contribute to the improvement of global visual health.

### 2 Photothermal treatment mechanisms and nanomaterial properties of ophthalmic nanomedicines

Nanomaterials with highly tunable photothermal conversion properties are referred to as photothermal nanomaterials [22]. These materials are categorized into three distinct types based on their different photothermal conversion mechanisms, which arise from their unique electronic structures [23–24]. The types include metals exhibiting localized surface plasmon resonance (LSPR), carbon and polymer materials undergoing molecular thermal vibration, and inorganic semiconductor materials that absorb light through bandgap transitions [25]. The specific photothermal properties of these materials, encompassing aspects such as range and rate of light absorption, photothermal conversion efficiency, heat transfer capability, and photothermal stability, play a pivotal role in determining their therapeutic mechanisms, the range of suitable therapeutic applications, and overall therapeutic effectiveness [26–27].

The selection of appropriate incident light has a significant impact on the effect of photothermal therapy. Based on wavelength, incident light can be divided into ultraviolet (UV; 190–400 nm), visible light (400–780 nm) and near-infrared light (NIR; 780–2500 nm) [28–29]. UV light has shorter wavelengths and higher photon energies that can be absorbed by most body tissues. Hence, its penetration is limited, and it may trigger a number of photochemical reactions [30–31]. UV light helps the skin synthesize vitamin D, but excessive exposure to UV light may lead to DNA damage, sunburn, and photochemical damage [32–33]. Visible and NIR light with longer wavelengths have lower photon energies and are safer for use in the human body [34]. The endogenous chromophores (i.e., blood, water, and melanin) and biomolecules in the human body have a low absorption rate of NIR light. Hence, NIR has a high tissue penetration depth, which can avoid photothermal damage to healthy tissues [35–37]. Activation of photothermal nanomaterials with NIR light selectively limits the photothermal conversion processes to the target tissue area, resulting in highly efficient, precise, and safe treatments [38–39].

#### 2.1 Plasmonic metal nanoparticles

**Figure 2:**
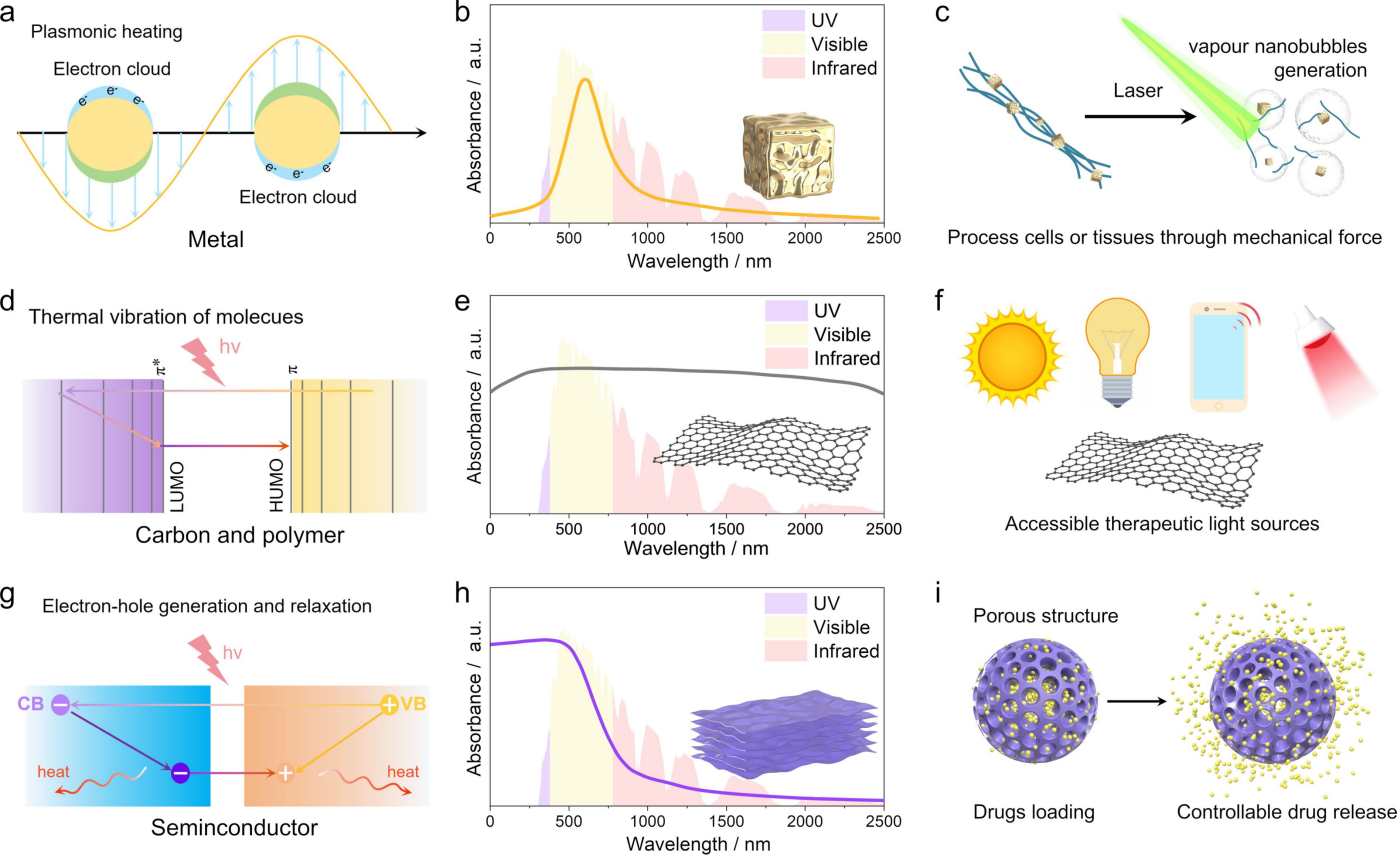
Exploring the photothermal conversion mechanisms and therapeutic applications of photothermal nanomaterials [92]. This illustration delineates the photothermal conversion mechanisms, properties, and therapeutic mechanisms of various photothermal nanomaterials. (a–c) Metals: These materials initiate photothermal conversion through localized surface plasmon resonance (LSPR), characterized by absorption at a single wavelength [48–50]. The therapeutic process involves mechanical forces generated by the rupture of vapor nanobubbles, effectively treating tissues or cells. (d–f) Carbon/polymer materials: These materials undergo photothermal conversion via molecular thermal vibration, displaying full-spectrum absorption [55–60,65–66]. This broad absorption range reduces the energy and diversity requirements for therapeutic light sources, enhancing the versatility of treatment applications. (g–i) Semiconductor materials: These materials leverage bandgap transitions for photothermal conversion, primarily absorbing short wavelengths. Their unique properties enable synergistic therapies combining photothermal effects with drug delivery, facilitated by their porous structures [72–81,85–88].

The vibrant colors of metals are indicative of their tendency to absorb light at single wavelengths, rather than across the full spectrum [40]. This phenomenon occurs as the electromagnetic field of incident light induces forced oscillations in the free electrons on the metal surface [41–42]. When the frequency of the incident light aligns with the intrinsic oscillation frequency of these surface electrons [43], it triggers a rapid, collective resonance among them [44]. This resonance leads to interactions between the excited free electrons and other electrons, lattice phonons, and surface ligands, converting the kinetic energy of these electrons into thermal energy through the Joule mechanism, an exceptionally efficient process with photothermal conversion nearing 100% efficiency (see below in Figure 2a) [45–47]. The specific absorption wavelength of these metals is closely linked to their extinction cross section and particle size and shape, which are greatly influenced by the chemical capping agents and the dielectric environment present during their synthesis (see below in Figure 2b) [48–49].

Because of the direct occurrence of photothermal conversion on the surfaces of LSPR metals and its rapid nature, combined with the metals’ inherent high thermal conductivity and strong hydrophilicity, LSPR nanometals can be swiftly heated to temperatures around 100 °C using low-energy laser pulses of specific wavelength. This rapid heating effectively evaporates a limited amount of water in the adjacent nanoscale region, forming vapor nanobubbles (VNBs) (see below in Figure 2c) [50]. The swift expansion and collapse of these VNBs transform thermal energy into mechanical forces, such as jets and acoustic shock waves, enabling cellular or tissue treatment with minimal thermal damage. Currently, VNBs are being explored for applications in cancer cell eradication [51], harmful protein aggregate degradation [52], and overcoming barriers in drug delivery [53].

Furthermore, the precise adjustment of the morphology and particle size of LSPR metals allows for the fine-tuning of their characteristic absorption within the range of 650 to 1350 nm. This range falls outside the absorption spectrum of the eye’s aqueous biological tissues, thereby minimizing photothermal damage to non-pathological areas [54].

#### 2.2 Carbon and polymer materials

Carbon and polymer materials primarily undergo photothermal conversion through a mechanism known as molecular thermal vibration. This process is initiated when the energy of incident photons aligns with the intramolecular electron orbital transition (π→π*) [55], resulting in electrons being excited from their ground state to higher energy orbitals [56]. As these excited electrons relax back to their ground state, they induce vibrations in the molecular lattice, which in turn release heat (see below in Figure 2d) [57]. The presence of a high density of loosely bound electrons and the narrow energy level spacing of the π electrons endow carbon materials (such as graphene, carbon nanotubes, carbon quantum dots, and fullerenes) and polymer materials (like polydopamine, polyaniline, and polypyrrole) with a broad light absorption spectrum and efficient photothermal conversion capabilities (see below in Figure 2e) [58–60].

In addition to polymer-based photothermal nanomaterials, organic small molecule dyes that are often used for tissue staining can also be used as photothermal agents [61]. Organic small molecule dyes are easy to remove from the eye, however, they suffer from low photothermal conversion efficiency, easy photobleaching, low water solubility, and low stability [39]. Common organic small molecule dyes include cyanine dyes (e.g., indocyanine green (ICG)), porphyrin dyes, rhodamine dyes, and squaraine dyes [62]. By modifying, adding, or removing functional groups in the molecule, the light absorption spectrum of organic small molecule dyes can be effectively adjusted, and targeting can be achieved. The photothermal conversion efficiency of organic small molecule dyes can be modulated by intramolecular rotation or intermolecular interactions [63]. By using nanocarrier encapsulation and self-assembly strategies, the water solubility and stability of organic small molecule dyes can be improved, and photobleaching can be reduced [64].

Materials that undergo molecular thermovibrational processes, with an ability to absorb light across the full spectral range, reduce the dependence on specific therapeutic light sources. This versatility even permits the utilization of sunlight or electronic screens to modulate drug release or to stimulate lacrimal glands for the treatment of dry eye syndrome (see below in Figure 2f) [65–66]. The abundance of surface functional groups in these materials provides an excellent chemical foundation for constructing therapeutic platforms. Furthermore, carbon and polymer nanomaterials offer several advantages for ophthalmic applications, including exceptional biocompatibility, biodegradability, wide availability, low cost, and a highly tunable structure, making them well-suited for such uses [67–71].

#### 2.3 Inorganic semiconductor materials

Inorganic semiconductor materials, such as TiO_2_, SiO_2_, and Fe_2_O_3_, possess conductivity levels that fall between those of conductors and insulators. Their light absorption characteristics are primarily determined by their bandgap width, ranging from 0 to 3 eV (Figure 2g) [72]. Semiconductors with narrow bandgaps are capable of absorbing incident light energy that is greater than or equal to their bandgap energy (incident wavelength from approximately 310 to 1240 nm) [73], leading to the generation of electron–hole pairs that possess energy equivalent to the bandgap [74]. Once these excited electrons are transferred to impurities, defects, or surface dangling bonds [75], they release energy via non-radiative relaxation, resulting in localized lattice heating [76–77]. Therefore, semiconductors with narrow bandgaps typically show broad absorption spectra and high efficiency in photon trapping. In contrast, wide-bandgap semiconductors have a more limited range of light absorption and less photothermal conversion capabilities. For instance, titanium dioxide (TiO_2_) with a bandgap of 3.3 eV, which is transparent to visible light, primarily absorbs ultraviolet light, and common semiconductor materials are only weakly absorbent in the NIR range (Figure 2h) [78].

In comparison to LSPR metals and carbon, semiconductor materials offer greater flexibility in photothermal property design because of a broader range of choices in chemical composition and crystal structure, coupled with well-established strategies for manipulating light absorption range through band engineering [79–82]. The bandgap width of TiO_2_ (≈3.3 eV) is relatively large; thus, absorption of visible light is very weak. Through non-metallic doping, some localized states can be generated above the O 2p orbitals, and the valence band of TiO_2_ can be reconstructed, resulting in an upward shift of the valence band and a narrowing of the bandgap, thereby enhancing visible light absorption [83]. For example, pristine TiO_2_ hardly absorbs visible light with wavelengths greater than 400 nm, while N-doped TiO_2_ quantum dots exhibit significant visible light absorption between 400 and 1000 nm [84]. Semiconductors that rely on ionic bonding are particularly well-suited for development into porous structures [85–86], which can significantly enhance their functionality. Porous semiconductor photothermal nanomaterials, characterized by their high specific surface area and pore volume, can be extensively loaded with drugs, facilitating controlled photothermal drug therapy (Figure 2i) [87–88]. Additionally, semiconductor materials are notable regarding their stability, ease of synthesis, and cost-effectiveness, making them highly promising for ophthalmic applications [89–90]. Some semiconductors with high surface carrier concentrations (e.g., TiO*_x_*, WO_3−_*_x_*, MoO_3−_*_x_*) can undergo photothermal conversion through LSPR, further enhancing their potential in generating VNBs [91].

### 3 Treatment of ophthalmic diseases with photothermal nanomaterials

#### 3.1 Ocular tumors

Ocular tumors significantly jeopardize both the vision and lives of patients, presenting a substantial challenge in simultaneously preserving life and sight [93–95]. Traditional treatment methods include enucleation, ocular chemotherapy, and radiation therapy [96]. Enucleation invariably results in permanent blindness, facial disfigurement, and potential psychiatric disorders [97]. Chemotherapy poses difficulties in accurately controlling drug deposition on the ocular surface and dosage, with frequent administrations often leading to complications such as retinal edema, vitreous hemorrhage, and ocular deformity [98–99].

Direct photothermal therapy (PTT) may induce irreversible thermal damage to surrounding tissues due to inadequate control over the heat distribution [100–105]. Innovations in this field have led to the development of transparent polylactic acid (PLA) thin films with embedded iron oxide nanoclusters prepared via spin coating [51]. The efficient photothermal conversion of iron oxide minimizes the necessary laser energy. The mechanical force generated by laser-induced VNBs enables the selective destruction of single corneal cells. The PLA films aid in precisely positioning the photothermal therapy, restrain the dispersion of iron oxide nanoclusters, and can be easily removed post-laser treatment. The spatially selective single-cell killing capability of iron oxide PLA bubble films has great potential for ocular tumor therapy. However, clinical treatment of tumors requires the application of films on surfaces with “odd” tissues. Using a sufficiently soft film to tightly cover the tissue can ensure direct contact between the VNBs produced by the film and tumor cells. The experimental results indicate that using more and higher-energy laser pulses produces more and larger vapor bubbles, which can kill cells at some distance from the film. A photothermal gel, composed of Au nanorods, geraniol, chitosan, and the gene-targeted drug DC_AC50 can be activated by NIR light. Photothermal activation softens the hydrogel composed of geraniol and chitosan, controlling drug release and facilitating PTT at moderate temperatures, thus yielding exceptional anti-tumor efficacy both in vitro and in vivo [106]. Additionally, Au nanoparticles synthesized using fucoidan (Fu-AuNPs) loaded with the chemotherapy drug doxorubicin (DOX), effectively inhibit choroidal melanoma via a synergistic PTT–chemotherapy approach [107]. Fu, as a reducing agent, assisted in the synthesis of AuNPs and served as a surface coating for AuNPs, promoting the coupling of DOX, enhancing anti-tumor activity, and improving the biocompatibility of AuNPs. The significant extinction coefficient of these nanoparticles enhances the contrast in photoacoustic imaging within the tumor region, aiding in the precise identification of treated areas. Au nanorods, when combined with anti-epithelial cell adhesion molecule (EpCAM), accurately target EpCAM+Y79 retinoblastoma cancer cells [108]. The targeted cells are deemed to be destroyed by VNBs induced by optimally parameterized femtosecond circularly polarized laser pulses, drastically reducing cell viability to about 10%. This targeted approach ensures that the laser energy remains below the threshold that could damage healthy cells, and the thermal field is efficiently confined to a 10 nm range around the cancer cells, thereby sparing adjacent healthy cells [109]. After eliminating tumor cells, small gold nanorods can penetrate the blood–brain barrier and be effectively excreted from the body through renal excretion, thereby avoiding the production of VNBs near non-cancerous cells.

#### 3.2 Posterior capsule opacity after cataract surgery

**Figure 3:**
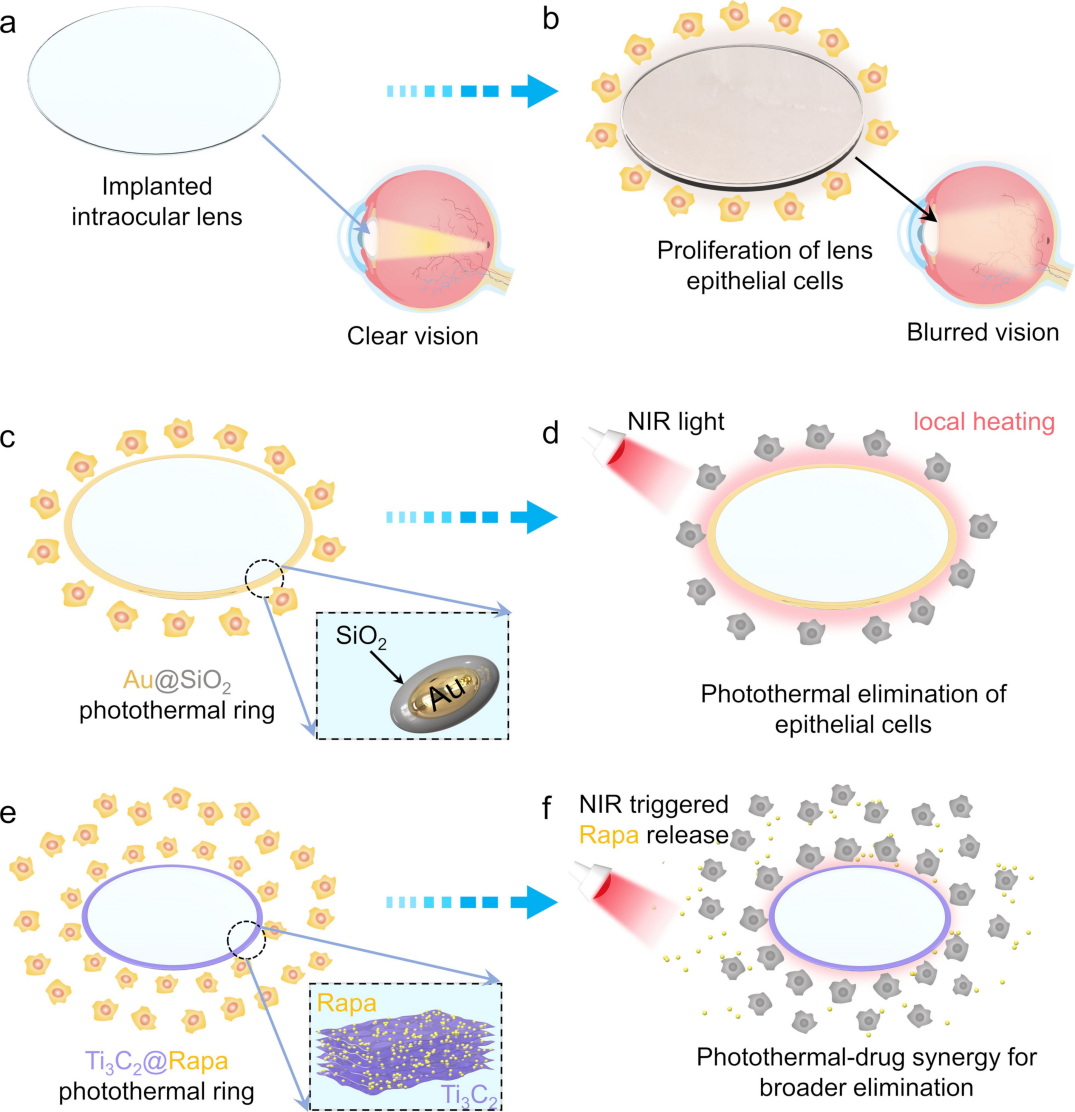
Application of photothermal nanomaterials in constructing edge photothermal rings for IOLs in the treatment of PCO. (a, b) Post-cataract surgery complications often involve the rapid proliferation, migration, and fibrosis of residual lens epithelial cells (LECs) around implanted intraocular lenses (IOLs), obscuring the visual axis and leading to potential blindness [111]. (c, d) The implantation of Au@SiO_2_-coated IOLs into eyes with cataracts in rabbits demonstrates the inhibition of LEC fibrosis. This is achieved through targeted photothermal treatment in specific areas under NIR irradiation, effectively addressing the issue of PCO [115]. (e, f) Ti_3_C_2_@Rapa-coated IOLs offer a synergistic approach in preventing PCO, combining the benefits of photothermal therapy with controlled release of the drug rapamycin (Rapa) under NIR light, showcasing a novel and effective strategy in PCO management [117].

Posterior capsule opacity (PCO) ranks as one of the most prevalent complications following cataract surgery (Figure 3a) [110]. This condition arises from the rapid proliferation, migration, and fibrosis of residual lens epithelial cells (LECs) that remain on the lens capsule, eventually leading to a progressive loss of vision (Figure 3b) [111–112]. The primary treatment for PCO currently is precisely focused capsulotomy [113]; however, this approach carries risks of severe complications, including retinal detachment and cystoid macular edema [114]. Zhu et al. [115] developed a photothermal ring made of silica-coated Au nanorods (Au@SiO_2_) placed at the edge of a commercially available intraocular lens (IOL) (Figure 3c). The localized photothermal conversion of Au@SiO_2_ effectively eradicated the residual LECs around the IOL using a low-energy laser (3.3 W·cm^−2^), thereby preventing disorganized fibrosis of LECs (Figure 3d). Consequently, the incidence of PCO in a rabbit model was approximately one third of that observed in the control group. Furthermore, photothermal nanomaterials with a wide absorption wavelength, such as reduced graphene oxide (rGO) [67] and polydopamine [68], have been utilized to construct IOL edge photothermal rings, showing promising results in inhibiting PCO.

The fixed heating removal area of the IOL edge photothermal ring, however, did not effectively inhibit LEC growth beyond the ring’s immediate vicinity. Employing black phosphorus (BP) as an IOL edge photothermal ring, coupled with the controlled release of DOX, resulted in a significant reduction in PCO incidence, that is, only 28% in a rabbit model, 100 days post-surgery, through a combination of photothermal and drug therapy [116]. Additionally, two-dimensional Ti_3_C_2_ nanosheets loaded into IOLs were used for a combined photothermal and chemotherapy treatment of PCO (Figure 3e) [117]. The ultrathin, planar structure of Ti_3_C_2_ provided numerous anchor sites for the drug rapamycin (Rapa), achieving a high loading capacity (92%). In a chinchilla rabbit model of PCO, NIR-triggered release of Rapa curtailed the migration of LECs and their inflammatory response following photothermal treatment, without causing significant damage to the surrounding healthy tissues (Figure 3f).

#### 3.3 Vitreous turbidity

**Figure 4:**
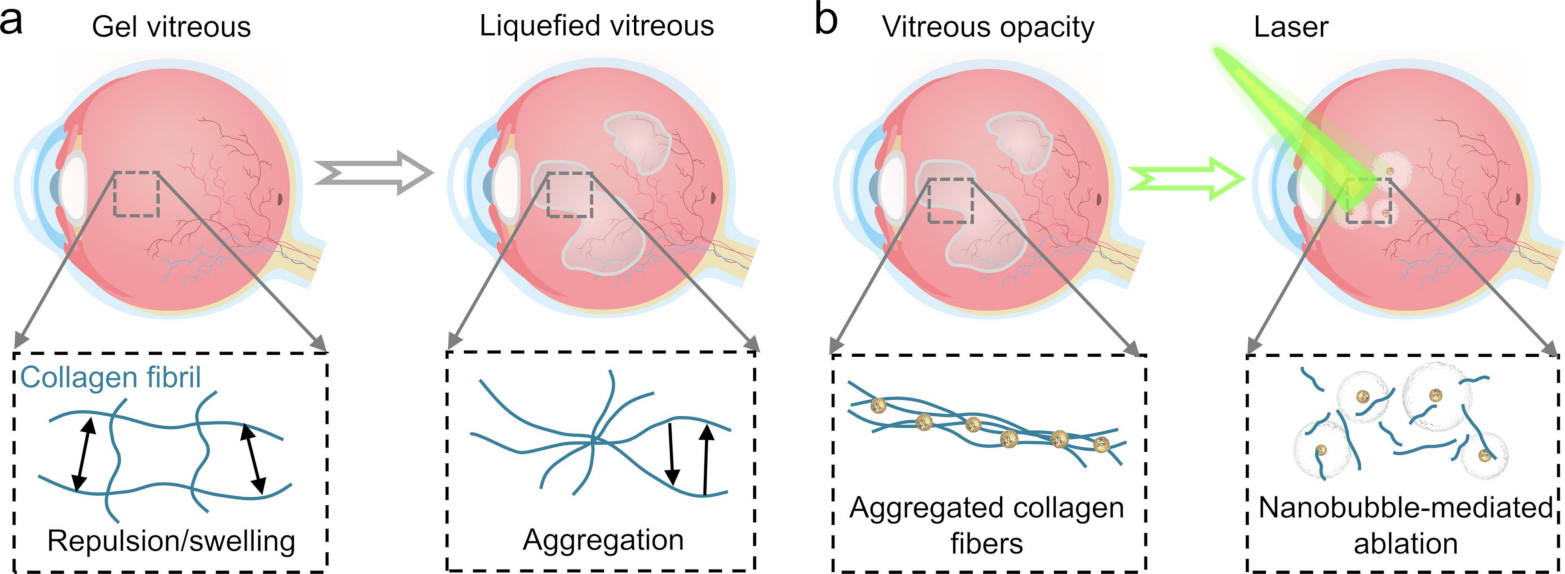
Mechanisms and treatment of vitreous opacity. (a) Vitreous gel liquefaction leads to repulsion, swelling, and subsequent aggregation of collagen fibers, culminating in vitreous opacity [119]. (b) Therapeutic approach in which VNBs, induced by pulsed laser irradiation of Au nanoparticles, mechanically disrupt opaque collagen fiber aggregates. This method effectively treats vitreous turbidity while minimizing thermal damage [122].

The vitreous is a highly hydrated, transparent gel supported by a network of long and thin collagen fibers [118]. Factors such as aging, myopia, or diabetes can lead to the liquefaction of this vitreous gel and the accumulation of collagen fibers, resulting in turbidity that casts shadows on the retina, manifesting as floaters in a patient’s field of vision (Figure 4a) [119–120]. Vitreous opacity can diminish contrast sensitivity and potentially contribute to impaired vision quality, adversely affecting the patient’s quality of life and leading to psychological issues [121].

Hyaluronic acid (HA)-coated AuNPs accumulate on human vitreous opacities obtained through vitrectomy [52]. When exposed to nanosecond low-energy laser pulses (about 1000 times weaker than the pulses used in standard clinical YAG laser therapy), these nanoparticles rapidly heat up, producing rapidly expanding and collapsing VNBs. This action generates jets and high-pressure shockwaves that mechanically disrupt the collagen aggregates in the vitreous. This method provides a safer and more effective approach for treating opacities near the retina (Figure 4b). Compared with HA-AuNPs (10 nm) that absorb light near 520 nm, the light absorption of the organic dye ICG mainly occurs in the NIR region, with less absorption of NIR by biomolecules, which contributes to safer photothermal therapy. In subsequent studies, after injecting exogenous collagen opacities into the vitreous of rabbit eyes for five days, free ICG was injected, and it was found that ICG could bind to the opacities. Afterwards, laser pulses were used to completely ablate the injected collagen opacities [122]. Cationic carbon quantum dots, known for their robust light absorption across a broad spectrum of wavelengths, have also been proven effective in breaking down collagen fibers and vitreous opacities through the VNBs generated by low-flux pulsed lasers [69,123].

#### 3.4 Glaucoma

**Figure 5:**
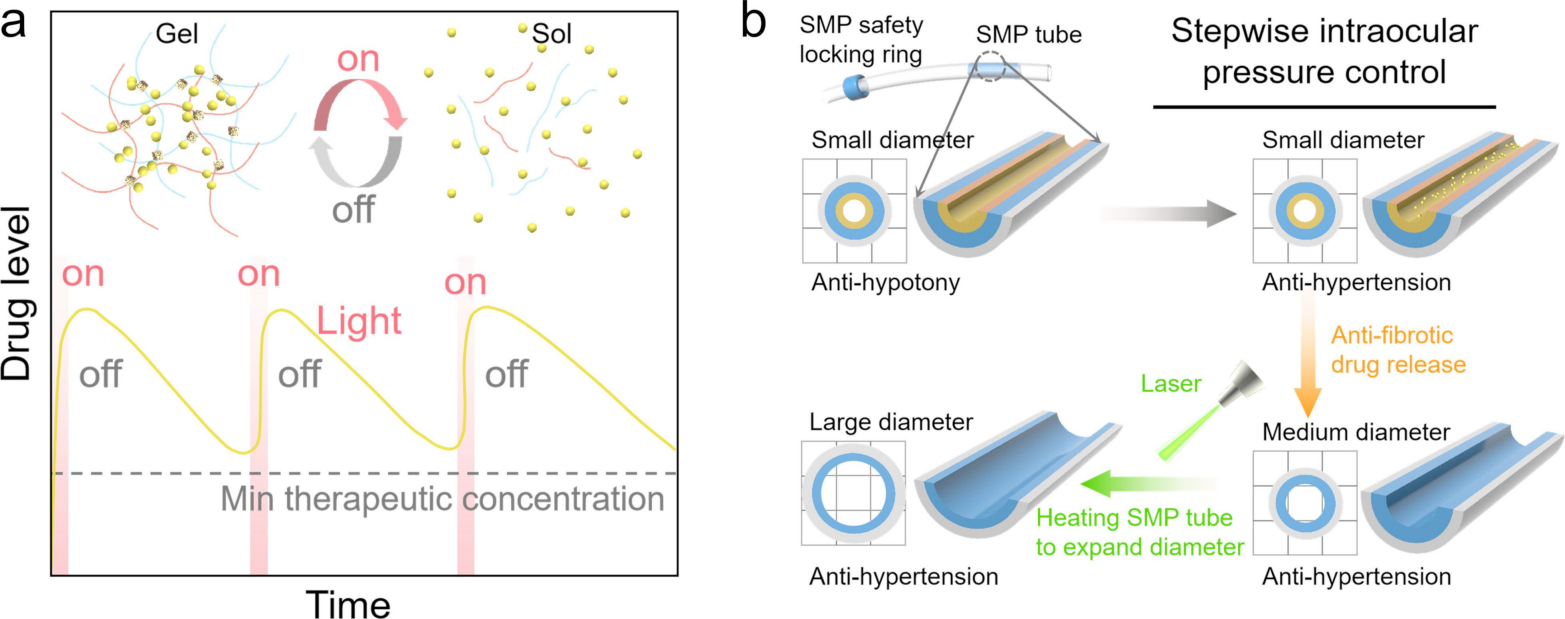
Utilizing photothermal nanomaterials in glaucoma treatment. (a) The heating of photothermal nanoparticles embedded within a hydrogel molecular network can cause the hydrogel to reversibly soften. This reaction facilitates the triggered, controlled release of drugs preloaded into the hydrogel [131]. (b) Application of laser heating to shape memory polymer tubes. This technique allows for precise adjustment of the tube’s diameter, thereby enabling a gradual and controlled regulation of intraocular pressure [133].

Glaucoma stands as the second most common cause of blindness globally. It arises from impaired circulation of aqueous humor, leading to an increase in intraocular pressure (IOP) [124]. This elevation in IOP can result in the progressive death of retinal ganglion cells and subsequent optic neuropathy [125–126]. Currently, ocular drug delivery is the prevalent treatment approach; however, it faces challenges due to drug degradation and obstacles in drug diffusion, rendering the therapy less effective [127–128]. Therefore, ocular drug delivery systems capable of controlled and sustained drug release are crucial to enhancing drug utilization efficiency and positively impact patient compliance [129–130]. Hydrogels that exhibit reversible gel-to-sol transitions upon heating are emerging as promising materials for photothermal drug release [131]. The application of visible and NIR light locally heats the photothermal nanomaterials embedded in these hydrogels, causing them to soften reversibly and release the encapsulated drug. The rate of drug release can be finely tuned by adjusting the concentration of the hydrogel and photothermal nanomaterial, as well as the irradiation conditions [132]. This method of controlled and sustained drug release holds significant potential for delivering drugs with limited half-lives and treating chronic ocular diseases (Figure 5a).

Most platforms for photothermally controlled drug release primarily focus on the delivery of a single drug [134–135]. Drawing inspiration from lollipops, Wang et al. developed a multilayered sodium alginate–chitosan hydrogel sphere drug delivery system, which uses ZnO-modified biocarbon (ZnO-BC) to enhance the photothermal conversion performance [70]. The hydrogel ball is embedded under the conjunctiva through surgery. ZnO-BC can effectively absorb NIR radiation and convert it into heat, thus activating the hydrogel to generate sol–gel transformation, and achieving on-demand drug release. Drugs such as timolol maleate and levofloxacin were encapsulated within separate layers of the hydrogel, facilitating a sustained, stepwise release, effectively managing elevated IOP and preventing infection.

Silica gel devices, used for decades to drain intraocular fluid and control IOP, often have limitations because of their fixed tube diameters [136–137]. These devices struggle to adapt to fluctuations in IOP caused by the disease process, patient-specific hypotony, or high IOP resulting from tissue fibrosis [138–141]. To address this, photothermal shape memory polymers (SMPs) have been employed to program and control the inner diameter of the drainage tubes (Figure 5b) [133]. These SMP silica gel drainage tubes consist of three layers, namely, an outer silica gel drainage tube, a middle SMP tube, and an innermost gel layer loaded with antifibrotic drugs. Initially, the SMP tube exhibits a small inner diameter (≈50 μm), preventing early hypotonia. As the innermost gel layer undergoes hydrolysis, antifibrotic drugs are released into the eye’s medial side, enlarging the SMP tube to a medium diameter (≈200 μm). Argon laser heating can further expand the SMP tube to a larger diameter (≈250 μm), providing controlled adaptation to patient-specific hypertonicity. Additionally, a safety locking ring outside the SMP tube can be compressed under laser induction to significantly reduce the tube’s diameter, thus preventing late hypotony. This programmable IOP control functionality has been demonstrated in rabbit eyes.

Creating a new aqueous humor filtration sites through glaucoma filtration surgery can also reduce IOP [142–143]. However, over time, the excessive proliferation of conjunctival fibroblasts and remodeling of collagen around the vesicles can lead to the failure of the surgical effects [144]. The primary strategy to counter fibrosis has been the use of antimetabolic drugs, but this often leads to complications like filter vesicle leakage, bacterial endophthalmitis, and ocular hypotonia. Wang et al. developed a polyvinyl alcohol (PVA) hydrogel with enhanced photothermal, antimicrobial, and drug-delivery capabilities (PVA@rGO-Ag/5-Fu) [71]. Under NIR irradiation, the rGO-Ag component in the hydrogel effectively targets and thermally destroys conjunctival fibroblasts and invasive bacteria around conjunctival vesicles, while the 5-fluorouracil (5-Fu) inhibits fibrous responses in filter vesicles, thus achieving effective IOP reduction [145].

#### 3.5 Endophthalmitis

Endophthalmitis, commonly resulting from pathogenic infections, is a frequent complication following ophthalmic surgery [146–148]. While antibiotics remain the most effective treatment against microbial infections, their global overuse has led to the emergence of drug-resistant bacterial strains [149–151]. In response, Zhou et al. developed a photothermal therapy agent, AuAgCu_2_O-NS, which consists of a core of AuAg alloy nanospheres and a Cu_2_O shell, specifically designed for treating non-healing keratitis caused by drug-resistant bacterial infections [152]. This agent’s controllable photothermal effects, coupled with the release of Ag^+^ ions from the AuAg core, work synergistically to eradicate multidrug-resistant bacteria. Additionally, the release of Cu^+^ ions from the Cu_2_O shell aids in accelerating endothelial cell angiogenesis and fibroblast migration, thereby enhancing the wound healing process. Clinical assessments, including ophthalmic scores, wound closure rates, and histopathological analyses, have demonstrated that AuAgCu_2_O-NS effectively promotes re-epithelialization of the wound area and eradicates complex bacterial infections in diabetic mice. Further studies have incorporated the anti-inflammatory drug bromfenac sodium into AuAgCu_2_O-NS, using it to combat local bacterial infections and severe inflammation in a rabbit model of endophthalmitis [153]. Moreover, a gel comprising AgCu_2_O and ethylene diamine tetraacetic acid (EDTA) has been formulated, demonstrating therapeutic efficacy in treating fungal keratitis [154]. In in vitro antibacterial experiments, the minimum inhibitory concentrations of AuAgCu_2_O NS (808 nm, 0.75 W·cm^−2^, 10 min) and AgCu_2_O-EDTA (808 nm, 0.25 W·cm^−2^, 5 min) were similar. However, AgCu_2_O-EDTA required a lower laser energy density and shorter irradiation times, resulting in a milder photothermal effect.

#### 3.6 Enable near-infrared vision

**Figure 6:**
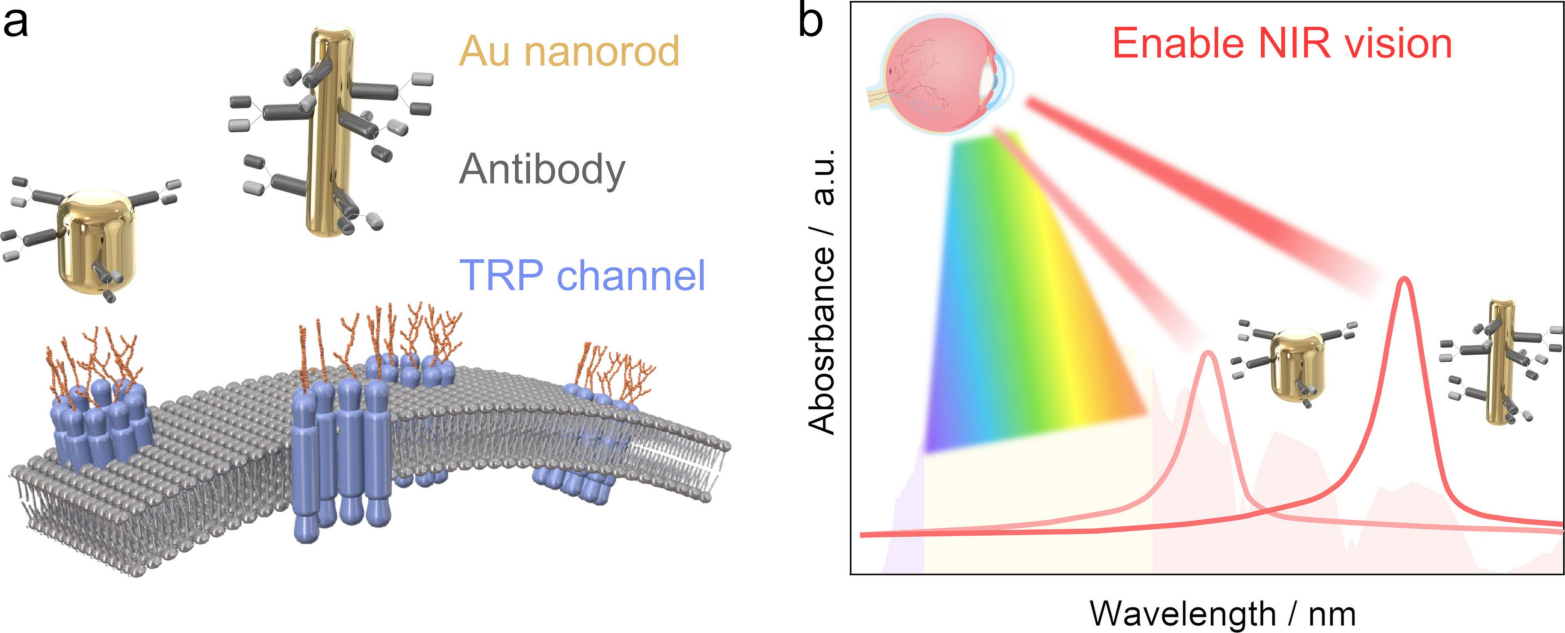
Enhancing NIR vision with photothermal nanotechnology. (a) Photothermal Au nanorods intricately coupled with temperature-sensitive ion channel antibodies. (b) Process of absorbing NIR light via Au nanorods, which facilitates targeted heating and activation of TRP channels, ultimately enabling the perception of NIR vision [160].

The human eye perceives visible light within the range of 380–780 nm, whereas certain species, such as pythons, possess the ability to detect infrared light (1000–30000 nm) using temperature-sensitive transient receptor potential (TRP) cation channels in specialized organs [155–157]. This capability allows them to overlay thermal and visual images, resulting in a more sensitive response to environmental changes [158–159]. Nelidova et al. [160] innovatively combined Au nanorods, known for their efficient absorption of infrared light, with temperature-sensitive ion channel antibodies. This combination successfully targeted the optic cone photoreceptors, inducing NIR photosensitivity in the residual photoreceptor cells of blind mice and human retinas tested in vitro (Figure 6a). By employing Au nanorods of varying lengths, the retina’s response to different NIR light wavelengths can be adjusted, and the response to various radiation intensities can be fine-tuned using engineered channels with differing temperature thresholds (Figure 6b). In mice, these NIR photosensitive photoreceptors activate cortical visual circuits, leading to behavioral responses. Remarkably, in human retinal samples obtained eight weeks post-mortem, NIR photo-responsiveness in the photoreceptor cells and their associated retinal circuits was reactivated. This groundbreaking research suggests that NIR vision, compatible with residual vision, can be enabled in blind retinas, representing a significant stride forward in enhancing human environmental perception capabilities.

#### 3.7 Facilitating drug delivery to the retina

The retina is the crucial visual tissue that converts light signals into nerve signals and transmits them to the brain. Functional damage of the retina often leads to progressive vision loss and even blindness [161]. Most retinal diseases still lack proper treatment, resulting in about estimated 196 million people worldwide suffering from age-related macular degeneration, 146 million from diabetic retinopathy, and 76 million from glaucoma [162]. Drug administration (primarily intravitreal, subretinal, and suprachoroidal injections) is a common strategy for alleviating retinal disorders; however, the ocular tissue barriers (primarily the vitreous, inner limiting membrane, retinal pigment epithelium, and blood–retina barriers) and defense mechanisms impede drug penetration and deposition [163]. Moreover, common ocular drug delivery methods have disadvantages such as short intraocular retention time, low drug accumulation, and low bioavailability [164].

Because of the transparency of the cornea and crystalline lens, the eye allows various wavelengths of light to penetrate, making photothermal nanomaterials particularly interesting for promoting drug delivery to the retina. Selective disruption of transmission barriers through photothermal action can promote drug delivery to the retina. Photothermally triggered delivery systems involve converting absorbed light energy into heat, which then triggers the release of the cargo from a heat-sensitive carrier [165]. Photothermal drug delivery systems allow for precise spatial and temporal control of drug release based on stimulus intensity and duration, thereby reducing the need for invasive ocular injections for the treatment of chronic ocular diseases, as well as the “explosive release” of passive drug delivery systems [116]. Furthermore, photothermal drug delivery systems can be surface-modified to prolong drug residence time, improve mobility, avoid trapping, and provide targeting capabilities, which helps to achieve customized treatment and reduce the required number of injections [106].

The inner limiting membrane (ILM) is a major obstacle preventing effective drug delivery to the retina after intravitreal injection [166]. Considering that the ILM is not necessary for adults, using photothermal nanomaterials to generate VNB ablation of the ILM covering the retina can help deliver drugs to the retina [167]. The common material used to generate VNBs, AuNPs, has disadvantages including long-term accumulated toxicity and fragmentation under laser irradiation [168]. ICG, which has been used in ophthalmology for clinical ILM staining, is better suited for generating VNBs on the ILM, and the high NIR absorbance of ICG is beneficial in the in vivo environment. Karen Peynshaert’s team [169] demonstrated that ICG can bind to the ILM and generate VNBs upon pulsed laser irradiation, thereby disrupting the bovine ILM and the unusually thick human ILM. In addition, this photoporation strategy allowed model nanoparticles to break through the ILM barrier for highly successful delivery to the retina and was also able to increase the efficacy of mRNA-loaded lipid nanoparticles in the bovine retina fivefold. In order to limit the ablation to the ILM more precisely, it is necessary to avoid ICG obstruction and trapping by the vitreous collagen network (the surface charge of ICG NPs should be negative or neutral, and the size of the ICG NPs should be less than 550 nm), as well as to avoid ICG crossing the ILM to reach the retina (the size of the ICG NPs should be greater than 100 nm).

Therefore, in subsequent studies, two types of ICG NPs were synthesized using lipids and poly (lactic-*co*-glycolic acid) (PLGA) [53]. Size, surface charge, and ICG concentration of the NPs were modulated by varying the synthesis conditions of the ICG NPs. Among them, only ICG liposomes synthesized using lipids can induce the production of VNBs. The ICG concentration in PLGA ICG NPs is below 15 μg·mL^−1^, which is not sufficient to rapidly increase the temperature to produce VNBs. Evaluation of the VNB effect in bovine retinal explants showed that ICG liposomes led to subtle disruption effects in the ILM, in which completely ablated ILM regions alternate with intact regions. Photoporation strategies to overcome the ILM have the potential to improve the efficacy of all retinal therapies impeded by ILM delivery barriers, including optogenetics [170], neuroprotection [171], retinal regeneration [172], and cellular reprogramming [173].

#### 3.8 Photothermal nanomaterials for ocular photoacoustic imaging

Advanced ophthalmic imaging techniques such as photoacoustic imaging (PAI), optical coherence tomography (OCT), OCT angiography (OCTA), fluorescence imaging (FI), scanning laser ophthalmoscopy (SLO), and fundus photography have significantly changed the diagnosis and monitoring of ocular diseases [174–176]. OCT is the most prevalent ophthalmic imaging technique, providing high-resolution and high-sensitivity imaging; however, the test penetration depth is only a few millimeters, which does not provide clear imaging of deeper ocular structures [177–178]. Fluorescence imaging is hindered by contrast agent photobleaching and phototoxicity, resulting in low image quality and biological side effects [179–180].

Photoacoustic imaging has deep penetration imaging capability and is sensitive to the neovascular system and irregular vascular networks (blood contains the endogenous PAI contrast agent hemoglobin) [181–183]. The working principle of PAI is to use a short-pulse laser to irradiate eye tissue or contrast agent, resulting in rapid localized warming, which triggers transient thermoelastic expansion. The ultrasonic signal generated by the expansion can be detected by an ultrasonic imaging transducer to provide deep structural images (5 μm to 10 cm) of the eye [184–186]. In addition, PAI can provide functional information, for example, blood flow, oxygen saturation, oxygenated and deoxygenated hemoglobin, oxygen metabolism, and melanin concentration [187–188]. The main limitations of PAI are the slower imaging speed and the decreasing resolution as the depth of imaging is increased [189].

The use of contrast agents can improve the imaging quality of PAI [190]. Moreover, with the help of contrast agents, more pathological molecular information can be provided [191]. However, small-molecule contrast agents commonly used in the clinic are limited by photobleaching, low photothermal conversion efficiency, and fast clearance [189,192]. Photothermal nanomaterials with high extinction coefficients that enhance photothermal-acoustic conversion are particularly suitable for PAI, helping to improve image resolution, enhance signal strength, and increase contrast [193–194].

AuNPs with excellent photostabilization are common PAI contrast agents [195–196]. Gold nanorods [197], gold nanostars [198], hollow gold nanocages [199], chains of gold nanoparticles [200], and ultraminiature chain-like gold nanoparticle clusters [201] have been used for the detection of ocular structures such as retinal blood vessels, choroidal neovasculature, and uveal melanomas, showing enhanced contrast and resolution of photoacoustic images [202]. Surface modification and control over shape and size can alter the LSPR characteristics of AuNPs, resulting in a shift in the light absorption peak. Matching pulsed laser wavelength and LSPR peaks helps to enhance the photoacoustic signals and minimize the laser-induced photothermal and photochemical damages [203].

Gels with internally embedded AgCu_2_O nanoparticles were applied for the treatment of fungal keratitis, and the residence time of the nanomedicine and the corneal therapeutic effect were monitored by dual-peak imaging with PAI and OCT [154]. The drug could be gradually eliminated from the body within 48 h, and almost no signals of corneal neovascularization were observed after AgCu_2_O NPs gel treatment; the cornea was thin and normal. The control group showed visible signals of developing corneal neovascularization and a thicker cornea due to inflammation and edema. A bimodal PAI system consisting of exogenous contrast agents (AuPt-ICG [204], R-s-ICG [205]) combined with endogenous contrast agents (hemoglobin [206]) helps to enhance photoacoustic imaging. The system allows for real-time assessment of vascular changes driven by anti-choroidal neovascularization (CNV) therapy and enables drug tracking, providing information on drug enrichment and residuals. After binding to vascular targeting molecules in vivo, exogenous contrast agents can achieve effective photothermal elimination of CNV through safe laser irradiation. Delivering stem cells to the trabecular meshwork to regenerate tissue and restore its function can treat glaucoma [207]. Labeling stem cells with the PAI contrast agent AuNSs allows for real-time monitoring of stem cell delivery and circulation in the anterior chamber, providing additional histological information. In addition to applying the photoacoustic effect to photoacoustic imaging, laser-induced focused ultrasound can also be used to perform high-precision cavitation ablation treatments of ocular tissues [208].

## Conclusion

Research on photothermal nanomaterials has opened new avenues in the treatment of various ophthalmic diseases, such as ocular tumors, vitreous opacity, posterior lens capsule opacity, glaucoma, and endophthalmitis. These nanomaterials, known for their precision in targeting, confined thermal fields, compatibility with a wide range of light sources, high adjustability, and ease of access, have paved the way for precise and controllable photothermal therapies. Notably, therapeutic platforms that integrate photothermal nanomaterials with drugs, antibodies, liposomes, hydrogels, heat/pH-sensitive materials, and shape memory materials have demonstrated therapeutic effects that excel traditional thermal therapies. The studies reviewed herein underscore the promising clinical potential of photothermal nanomaterials in ophthalmology (Table 1). Presently, research in this field remains predominantly in the conceptual validation phase, but it holds the promise of developing advanced treatment technologies for a broader spectrum of ophthalmic diseases in the future.

**Table 1:** Photothermal nanomaterials for the treatment of ophthalmic diseases.

Ophthalmic diseases	Photothermal therapy platform	Therapeutic mechanisms	Therapeutic effect	Ref.

ocular tumors	transparent polylactic acid films loaded with Fe_2_O_3_ clusters	VNB ablation	single-cell killing of bovine cornea	[51]
	hydrogels composed of Au rods, gene-targeting drugs, geraniol and chitosan	nanoparticle PTT, photothermally controlled drug release	effective treatment of uveal melanoma	[106]
	Au nanoparticles coupled with doxorubicin	nanoparticle PTT, photothermally controlled drug release	effective treatment of choroid melanoma	[107]
	Au nanorods coupled with anti-epithelial cell adhesion molecules	VNB ablation	EpCAM+ Y79 retinoblastoma viability decreased to ≈10%	[108]
	Au nanoparticles	VNB ablation	effective treatment of floating clusters of Y79 retinoblastoma cells	[109]
posterior capsule opacity	Au@SiO_2_ photothermal ring	nanoparticle PTT	incidence of PCO reduced to one third	[115]
	rGO photothermal ring	nanoparticle PTT	preventing PCO in rabbit models	[67]
	polydopamine photothermal ring	nanoparticle PTT	effectively inhibits PCO	[68]
	black phosphorus loaded with doxorubicin photothermal ring	nanoparticle PTT, photothermally controlled drug release	incidence of PCO reduced to 28% 100 days after surgery	[116]
	Ti_3_C_2_ loaded with rapamycin photothermal ring	nanoparticle PTT, photothermally controlled drug release	effectively inhibits lens epithelial cell migration and inflammatory response	[117]
vitreous opacity	Au nanoparticles coated with hyaluronic acid	VNB ablation	ablated collagen aggregates in rabbit eyes and patient vitreous specimens	[52]
	carbon quantum dots	VNB ablation	effectively ablated collagen fibers in the vitreous opacity	[69]
glaucoma	hydrogel loaded with ZnO-BC, timolol maleate and levofloxacin	photothermally controlled drug release	effectively controlled high intraocular pressure and prevented infection	[70]
	photothermally responsive shape memory polymers	reversible thermally induced deformation of shape memory polymers	satisfied the demands for intraocular pressure control at different stages	[133]
	PVA hydrogels loaded with rGO-Ag and 5-Fu	nanoparticle PTT, photothermally controlled drug release	effectively reduced intraocular pressure	[71]
endophthalmitis	AuAgCu_2_O-NS	nanoparticle PTT and photothermally controlled drug release	promoted re-epithelization of the wound area and eliminated bacterial infection	[152]
	AuAgCu_2_O loaded with bromfenac sodium	nanoparticle PTT, photothermally controlled drug release	eliminated bacterial infections and severe inflammation	[153]
	EDTA gels loaded with AgCu_2_O	nanoparticle PTT, photothermally controlled drug release	treated fungal keratitis	[154]
enable NIR vision	Au nanorods coupled with TRP antibodies	activation of TRP channels by Au nanorods	NIR photosensitivity was induced in the remaining photoreceptor cell of blind mice and in the vitro human retina	[160]
macular degeneration	agarose hydrogels loaded with bevacizumab and Au nanoparticles	photothermally controlled drug release	effectively controlled the release of biomacromolecule drugs	[131]
xerophthalmia	hydrogels loaded with Au@Pd nanorods	photothermal stimulation of the lacrimal gland	lacrimal glands are stimulated to produce more tears by light irradiation	[209]

**Figure 7:**
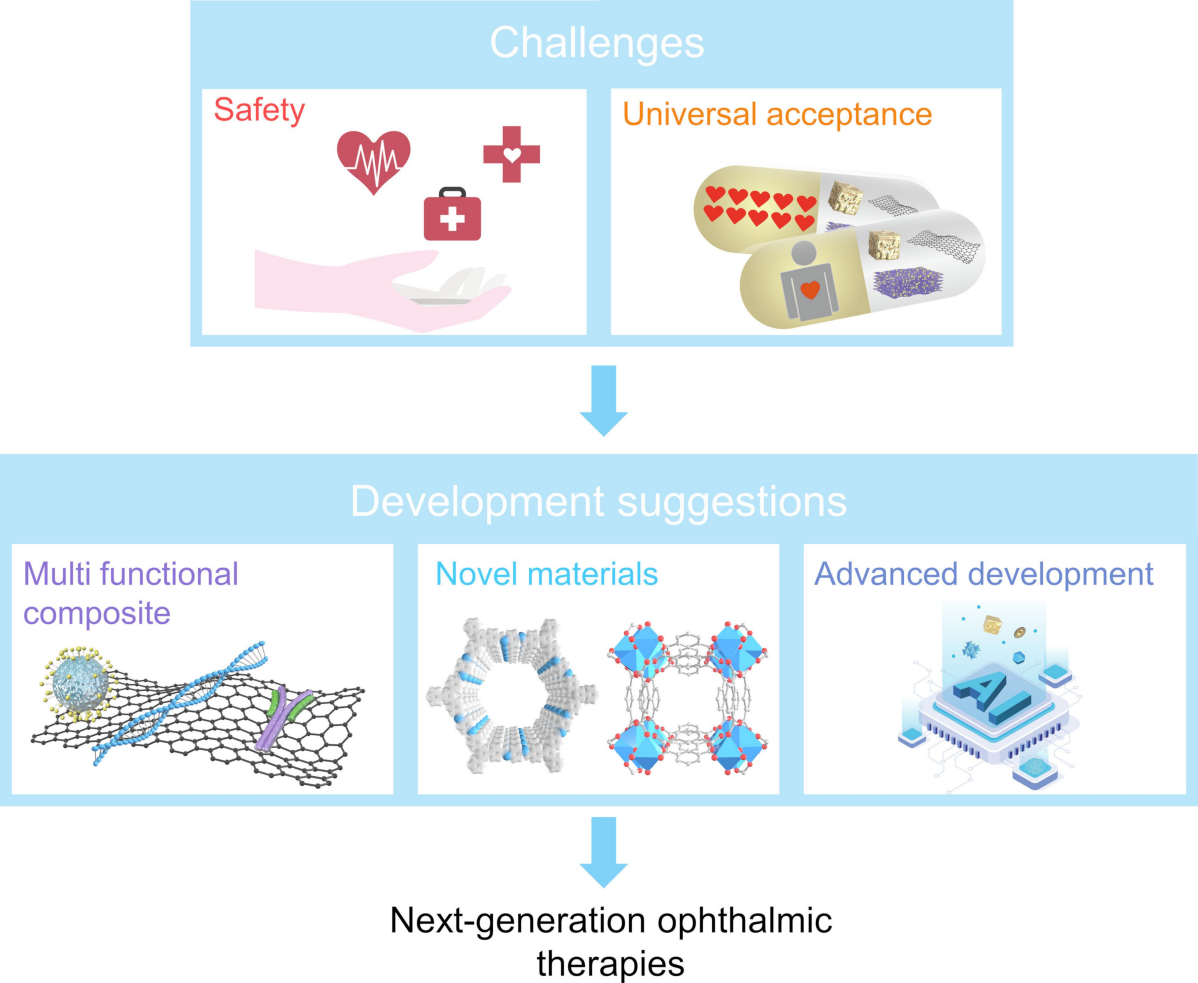
Prospects of photothermal nanomaterials for ophthalmic applications.

Photothermal nanomaterials have exhibited considerable potential in ophthalmic therapy, promising to overcome the limitations of current ophthalmic treatment technologies. By using machine learning to accelerate material development, exploring more types of photothermal nanomaterials, exploring more diverse composite photothermal material formulations, utilizing advanced characterization techniques, and collaborating with multidisciplinary researchers, more advanced and effective ophthalmic photothermal nanomaterial treatment methods will emerge in the future. However, several challenges need addressing before these technologies can be integrated into clinical ophthalmology (Figure 7).

Safety remains a paramount concern for the clinical application of photothermal nanomaterials [210–211]. Rigorous safety research, utilizing animal models, is essential to assess toxicology, pharmacokinetics, pharmacodynamics, and biological impacts [212]. It is crucial to consider the anatomical and physiological similarities between animal models and human eyes to accurately predict safety and therapeutic effects in humans [213]. The safety profile of photothermal nanomaterials is influenced by various factors, including their morphology, structure, concentration, photothermal stability, mechanical strength, and surface chemistry [214]. Enhancements in biocompatibility and stability can be achieved through coating processes or surface modifications. While structure and composition of new ophthalmic photothermal nanomaterials are often complex, understanding the safety of base materials can provide insights into the safety of more advanced derivatives. Safety discussions should include detailed explanations of material size, structure, dosage, and administration methods [215]. During treatment, processes such as nanoparticle aggregation, material degradation, cellular uptake/excretion, and unintended release of adsorbents require comprehensive safety analysis. In inorganic photothermal nanomaterials, because of the chemical inertness of Au atoms, AuNPs are widely used, especially for the generation of VNBs [216]. However, overly stable AuNPs cannot be biodegraded and can only be excreted metabolically from the body, and particles may fragment during rapid photothermal heating. Small-sized AuNPs can be excreted by penetrating the renal filtration barrier, but too small AuNPs may enter cell membranes and irreversibly bind to cellular biopolymers, leading to cytotoxicity [217]. Large AuNPs may accumulate in eye, liver, and spleen, causing long-term toxicity. Surfactants such as CTAB and CTAC used to assist in the synthesis of AuNPs or to prevent their aggregation may cause damage to DNA and cell membranes. Compared with inorganic nanomaterials, organic photothermal nanomaterials have the advantages of good biocompatibility, easy biodegradation, ease of modification, low cost, targeting, tunable light absorption, and relatively mild photothermal warming, which indicate great potential for clinical applications, especially for biomolecular polymers and ophthalmic organic dyes [218]. Moreover, by increasing the density of chromophores, organic photothermal nanomaterials are also able to produce VNBs effectively.

Research on composites of photothermal nanomaterials with other substances is currently limited, but promising therapeutic effects, especially when combined with biomolecules affecting cellular metabolism and function, have been observed. Therefore, further exploration regarding the modification and loading of photothermal nanomaterials is warranted to address more ophthalmic conditions. Modifying these materials with specific targeting molecules could extend their therapeutic applications to various eye tissues. Surface doping with metabolism-related ions could enable precise control over cellular functions. Future developments may see the integration of photothermal nanomaterial therapies with viruses, receptors, antibodies, aptamers, peptides, multifunctional genes, self-assembled DNA structures, and proteins. Given the diversity and adaptability of nanomaterials, it is conceivable to develop photothermal nanomaterial therapy systems tailored to individual patient conditions.

Currently, in the R&D stage of advanced mechanisms in the field of ophthalmic photothermal nanomaterials research, little attention has been paid to regulating light absorption and thermal management during design and selection. Additionally, because of concerns over material toxicity, novel materials like metal-organic frameworks, covalent organic frameworks, high-entropy materials, single-atom materials, electric power generation nanomaterials, 3D bioprinting materials, and upconversion luminescent materials have not been extensively explored. The use of such novel materials could significantly improve photothermal properties and contribute to therapeutic breakthroughs.

The traditional development of photothermal nanomaterials, often reliant on trial and error and experiential guidance, leads to lengthy development cycles and high cost. The rapid advancement in AI and machine learning is revolutionizing material design and screening processes [219]. Machine learning has achieved significant success in predicting various material properties, including morphology, toxicity, photothermal characteristics, synthesis methods, and activity [220]. Developing machine learning models for ophthalmic photothermal nanomaterials will expedite the development of high-performance target materials and alleviate the burden of extensive experimental work. Advanced characterization tools, theoretical simulations, and high-throughput testing techniques are expected to make substantial contributions to the advancement of ophthalmic photothermal nanomaterial development [221].

The evolution of photothermal nanomaterial technology represents just one facet of the multifaceted challenge posed by ophthalmic diseases. Comprehensive, patient-centered research regarding various ophthalmic conditions is crucial to ensure the widespread future use of photothermal nanomaterials. Economic, social, and cultural initiatives to enhance awareness about photothermal nanomaterials and their impact on healthcare policy, especially for underprivileged patients, are necessary for their affordability and acceptance. Photothermal nanomaterials hold immense promise for development, and it is anticipated that ongoing research will enable them to effectively address clinical ophthalmic diseases.

## Data Availability

Data sharing is not applicable as no new data was generated or analyzed in this study.
